# Assessment of the Hearing Status of School-Age Children from Rural and Urban Areas of Mid-Eastern Poland

**DOI:** 10.3390/ijerph18084299

**Published:** 2021-04-18

**Authors:** Edyta Pilka, W. Wiktor Jedrzejczak, Krzysztof Kochanek, Malgorzata Pastucha, Henryk Skarzynski

**Affiliations:** 1 Department of Experimental Audiology, Institute of Physiology and Pathology of Hearing, Mochnackiego 10, 02-042 Warsaw, Poland; w.jedrzejczak@ifps.org.pl (W.W.J.); m.pastucha@ifps.org.pl (M.P.); 2 Institute of Physiology and Pathology of Hearing, Mochnackiego 10, 02-042 Warsaw, Poland; k.kochanek@ifps.org.pl (K.K.); skarzynski.henryk@ifps.org.pl (H.S.)

**Keywords:** pure tone audiometry, otoacoustic emissions, TEOAE, tympanometry, hearing screening

## Abstract

(1) Background: The purpose of this study was to assess the prevalence of hearing loss in school-age children from rural and urban areas of mid-eastern Poland using standard audiological tests—pure tone audiometry (PTA), impedance audiometry (IA), and otoacoustic emissions (OAEs). (2) Methods: Data were collected from a group of 250 children aged 8 to 13, made up of 122 children from urban areas and 128 children from rural areas of mid-eastern Poland. Hearing was assessed in each of the subjects by means of PTA, IA (tympanometry), and transient-evoked OAEs (TEOAEs). Otoscopy was also performed. (3) Results: There were significantly fewer abnormal results in children from urban than rural areas: they were, respectively, 10.1% and 23.1% for IA, 3% and 9.7% for PTA, and 17.3% and 31.8% for TEOAEs. For hearing-impaired ears in rural areas (failed TEOAE), hearing thresholds were, on average, 11.5 dB higher at 0.5 kHz than for children in urban areas. Comparison of each PTA result with the corresponding IA showed that all cases of hearing loss were related to malfunction of the middle ear. (4) Conclusions: The results of all three hearing tests were significantly worse in children from rural areas compared to those from urban areas. This indicates that audiological healthcare in rural areas needs improvement and that universal hearing screening programs for school-age children would be helpful.

## 1. Introduction

According to the World Health Organization (WHO), over 6% of the world’s population has various types of hearing impairment [1]. This is a serious problem, regardless of the age at which it occurs, since hearing plays such an important role in communication. However, it is particularly serious for children, as it can inhibit linguistic development, which in turn gives rise to difficulties in communication, decreased cognitive function, and learning problems [2,3,4]. One effective solution for this issue is early intervention involving a prosthesis, which can improve the child’s quality of life in both audiological and non-audiological terms [5,6,7].

Unfortunately, access to hearing diagnostics is not equal everywhere. Healthcare in rural areas is definitely worse than in larger cities, both in Poland and in the rest of the world [8,9,10,11,12,13,14]. It is affected by various factors, including sociocultural, educational, economic (e.g., infrastructure), and even political issues [9,10]. 

This imbalance can be somewhat restored if a country has a universal newborn hearing screening (UNHS) program in place, by which all children, regardless of their place of residence, undergo a hearing test for detection of congenital hearing defects [15,16,17,18,19,20]. In some countries, tests are also done on school-age children in order to detect acquired hearing impairment; unfortunately, these programs are not universal and do not exist in most countries [21].

The data in the literature show that, for various reasons, the percentage of hearing impairment in school-age children is greater in rural areas than in built-up areas [22,23] and is most often associated with middle ear infection [24,25]. In young children from provincial southern India [23] and in some European countries [26,27,28,29,30], the percentage of hearing impairment exceeds 10%, while in Africa and Central Asia it can exceed 20% [29,30]. The percentage of hearing impairment for larger cities in Canada, India, and the United States ranges from about 8 to 15% [31,32,33]. In older children (12–15 years) living in the provinces of Russia or Europe, the percentage appears to be less than 10% [29,34]. In urban areas of Canada, India, and The Netherlands, the percentage of incorrect results in a similar age group is about 7% [31,32,35] and is slightly higher, 9–10%, in Poland and China [27,36]. Nevertheless, other studies from the United States and South Korea have shown that the percentage of incorrect results in older children living in a large city can exceed 20% [37,38]. This overview shows that hearing problems in children are substantial, while at the same time indicating that there is great variability in the available data. This points to the need for similar studies that might help define the problem more accurately.

Most of the cited studies have used screening audiometry, e.g., [22,27,33,38]. A few studies have assessed the patency of the ear canal [22,24,36], and others have used tuning forks [32], impedance audiometry (IA), or otoacoustic emissions (OAEs) [13,24,31,34,35]. To better understand the type of hearing loss and to explain regional differences in different countries, more data are needed, collected with the use of several test methods on the same subjects at the same time.

The aim of this study was to assess the prevalence of hearing loss in school-age children from rural and urban areas of Poland using standard diagnostic tests—pure tone audiometry, IA (tympanometry), and OAEs.

## 2. Materials and Methods

### 2.1. Participants

Measurements were performed in a group of 250 children (133 girls, 117 boys) aged 8 to 13 years (M = 10.41; SD = 1.75), made up of 122 children from urban areas (average age 10.24 years; SD = 1.9) and 128 children from rural areas (average age 10.59 years; SD = 1.6) of mid-eastern Poland (Masovian and Łódź Voivodships). For the final analysis, only the ears on which a complete test battery was performed were considered—392 ears in total. The dataset was divided into two groups: Group_u—197 ears of children from urban areas; and Group_r—195 ears of children from rural areas. In Poland, there is a clear administrative division into town (and city) and village which was the basis of classification for the urban and rural groups. In short, for a town to receive municipal rights, over 2000 people must be registered in it, there must be municipal buildings (not farm buildings), and at least two-thirds of the residents must be employed outside agriculture. Nevertheless, generally, by rural areas we mean areas characterized by smaller settlements (villages) and of an agricultural character, while by urban areas we mean areas with larger settlements (towns and cities) and industry.

### 2.2. Procedures

The tests were carried out as part of a hearing screening program of school-age children which was organized by our center [25,26,27]. However, in the group studied here slightly different procedures were used. The tests were carried out in the low-noise background of an isolated room within the school building (sound attenuation of the room was in accordance with the guidelines contained in the PN-EN ISO 8253-1/2005 standard and noise in the room did not exceed 20 dB SPL). The status of the ears of each subject was assessed using otoscopy (visual evaluation of the ear, mainly the tympanic membrane), impedance audiometry (IA), pure tone audiometry (PTA), and transient-evoked otoacoustic emissions (TEOAEs).

PTA was performed using the Madsen Astera clinical audiometer (GN Otometrics, Taastrup, Denmark). Air conduction hearing thresholds were determined for the frequency range 0.5 to 8 kHz using Sennheiser HDA-200 headphones (according to the National Institute for Occupational Safety and Health [39] the headphone attenuation values for individual frequencies are 0.5 kHz—35 dB, 1 kHz—42 dB, 2 kHz—46 dB, 4 kHz—53 dB, and for 8 kHz—55 dB). Hearing threshold was determined using the modified Hughson–Westlake method [40]. The child was regarded as having normal hearing if air conduction thresholds at all tested frequencies were 20 dB or less, based on the criterion developed by the International Bureau of Audiophonology (BIAP) [41].

IA in the form of tympanometry measurements was made using a Madsen Otoflex impedance bridge (GN Otometrics). A standard test tone of 226 Hz was used. The shape of the resulting tympanograms was assessed on the basis of the criteria proposed by Jerger and Liden in which there are 7 types of tympanograms (A, As, Ad, B, C, D, and E) [42,43]. Tympanograms classified as type B or C were considered abnormal. Children with type As tympanograms were included with those with type A tympanograms due to the fact that the results of other diagnostic tests for these cases did not show abnormalities. As noted by some authors, decreased compliance of the eardrum (resulting in the type As tympanogram) can also occur in healthy ears [44]. There were no subjects with type Ad, D, or E tympanograms.

The Otodynamics ILO 292 system (Hatfield, UK) was used to record TEOAEs. A broadband click of 80 μs at 80 ± 3 dB SPL (using the non-linear protocol) was used as stimulus, and a 20 ms analysis window was used. The recording was terminated after obtaining 260 averages. The record was assessed as ‘pass’ by taking account of the total repeatability parameter, which had to be higher than 70%, and the signal-to-noise ratio, which needed to be ≥3 dB SPL in at least three half-octave bands with center frequencies of 1, 1.4, 2, 2.8, and 4 kHz [45,46,47,48,49].

The child’s parents gave written informed consent. The research procedures were approved by the Ethics Committee of the Institute of Physiology and Pathology of Hearing, Poland (approval number IFPS: /KB/13/2014).

### 2.3. Data Analysis

For analyses we used Statistica v. 12 (StatSoft Inc., Tulsa, OK, USA). For all measured parameters, the statistical significance of mean differences was evaluated using analysis of variance (ANOVA). As a criterion of significance, a 95% confidence level (*p* < 0.05) was chosen. The figures were created in Matlab (version 2018b, MathWorks, Natick, MA, USA).

## 3. Results

Initial ANOVA analysis did not reveal differences in terms of gender (*F*(1.368) = 0.04; *p* = 0.85) or ear (left/right) (*F*(1.368) = 0; *p* = 0.99); therefore, all results are presented without taking these factors into account.

### 3.1. Otoscopy

Otoscopic examinations revealed middle ear abnormalities in 37 ears of children from rural areas and 17 ears of children from urban areas. Those with earwax were referred to a doctor for ear cleaning and invited for another hearing test.

### 3.2. Impedance Audiometry (IA)

Table 1 shows for Group_u and Group_r the frequency of occurrence of particular types of tympanograms. There were fewer incorrect tympanograms in urban areas (10.1%), while in rural areas there were almost twice as many (23.1%).

A two-way ANOVA revealed a significant effect of place of residence on the tympanometry result (*F*(1.380) = 5.7; *p* = 0.02). Furthermore, there was also a significant effect of age (*F*(5.380) = 3.7; *p* = 0.00) and interaction of place of residence and age (*F*(5.380) = 3.7; *p* = 0.003).

As can be seen from Table 2, children aged 11 and 12 from urban areas did not have any type B or C tympanograms (incorrect results), while in those aged 10 and 13 years there were also none who had type B. In Group_r (rural areas) in 8- and 13-year-olds there were no cases of excessively negative middle ear pressure (type C), and, similarly, in 10-year-olds no Type C tympanograms were found. The worst tympanometry results in Group_u were obtained in children aged 8, while in Group_r they were obtained in children aged 9.

### 3.3. Pure Tone Audiometry (PTA)

**Figure 1:**
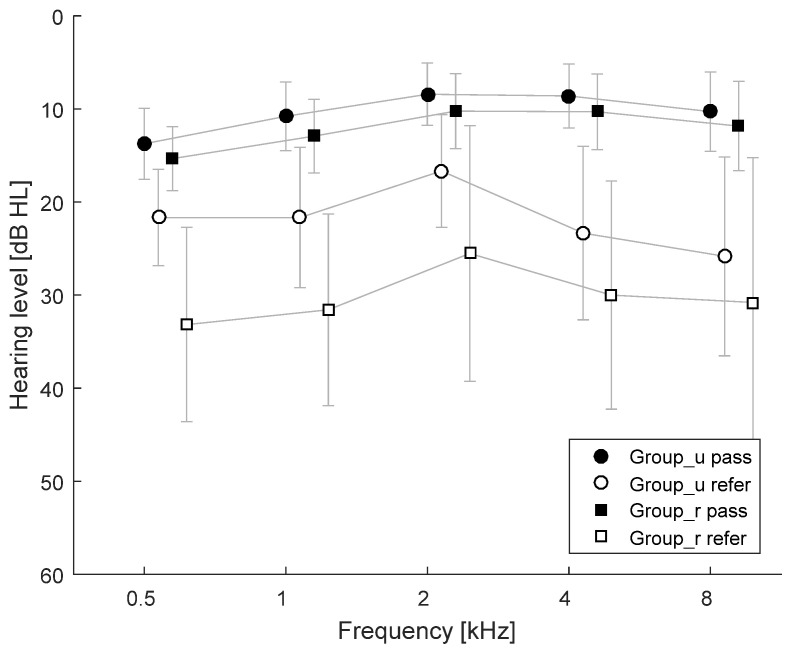
Average hearing thresholds for Group_u (urban) and Group_r (rural) divided into normal hearing (pass) and hearing loss (refer) subgroups.

Analysis of the PTA results showed that in Group_u abnormal hearing thresholds were obtained in 6 ears while in Group_r they were observed in 19 ears. Figure 1 shows the average hearing thresholds for both groups with a division into normal hearing (pass) and hearing loss (refer, i.e., a hearing threshold worse than 20 dB). On average, the hearing thresholds in impaired ears were 11.5 dB worse in children with Group_r (at 0.5 kHz) compared to children in Group_u. For all tested frequencies, the average difference was 8.4 dB. In impaired ears, the smallest difference in hearing threshold between the groups was 5.0 dB at 8 kHz.

In ears with normal hearing, the average difference between hearing thresholds for both groups was 1.8 dB across all tested frequencies, in favor of children from urban areas. The greatest difference was 2.1 dB at 1 kHz, and the smallest was 1.6 dB at 8 kHz.

Repeated measures ANOVA revealed an effect of place of residence on PTA (*F*(5.25) = 4.8; *p* = 0.0003), and also of age (*F*(5.25) = 1.8; *p* = 0.01), and there was also an interaction between place of residence and age (*F*(5.25) = 2; *p* = 0.002).

Table 3 presents the percentages of normal hearing thresholds and hearing loss by age and area of residence. As can be seen, in urban areas, children aged 10 to 12 years had no hearing loss in the examined ears, while in rural areas it occurred only in 11-year-olds. In Group_u, the highest percentage of hearing loss was found in 8-year-olds, and in Group_r it was in 9-year-olds.

### 3.4. Transient-Evoked Otoacoustic Emissions (TEOAEs)

**Figure 2:**
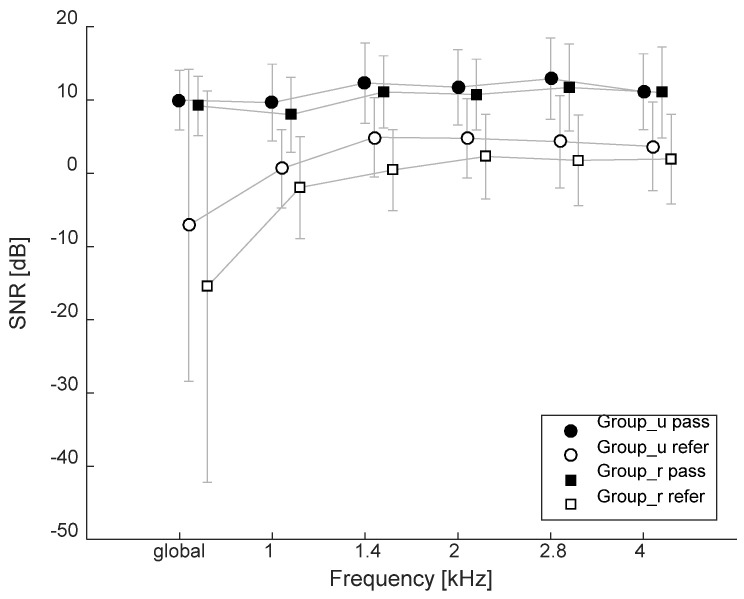
Average SNR values for TEOAE results for Group_u (urban) and Group_r (rural), broken down into those that met the OAE presence criteria (pass) and those that did not (refer). Data are shown for global (global = SNR for the whole broadband TEOAE signal) as well as for half-octave frequency bands.

Figure 2 shows the average SNR values for Group_u and Group_r, where “pass” means that the results presented in this group meet the criteria for the presence of responses described in more detail in the Methods section, and “refer” means they do not meet the criteria. In total, in both groups, the criterion for the presence of a response was not met in 96 ears, made up of 34 ears from Group_u and 62 ears from Group_r.

A two-way ANOVA showed an effect of place of residence (*F*(5.25) = 3.8; *p* = 0.002), effect of age (*F*(5.25) = 1.7; *p* = 0.02), and no interaction between place of residence and age (*F*(5.25) = 1.1; *p* = 0.3) on SNR.

Table 4 shows the percentage of TEOAE pass results according to age. As can be seen, the highest levels of “refer” findings were found in 12-year-olds (27.1%), and they were also high in 13-year-olds (20.3%) and 8-year-olds (21.9%). The lowest levels were in 10-year-olds (18%).

### 3.5. Summary of Results

Table 5 compares the number of ears with “refer” results. As can be seen, the highest number of “refer” results in both groups were obtained for OAEs, including 34 ears from Group_u (17.3%) and 62 ears from Group_r (31.8%). The lowest number of “refer” results were obtained for PTA—for 6 ears from urban areas and for 19 ears from rural areas. In terms of a “refer” result for any test, it applied to 38 of 197 ears in Group_u (19.3%) and 66 of 195 ears in Group_r (33.9%). 

## 4. Discussion

The aim of this study was to assess the prevalence of hearing loss in school-age children from rural and urban areas of Poland using standard diagnostic tests undertaken in everyday audiological practice—threshold examination by PTA, otoscopy, and IA. In order to increase the accuracy of the assessments, TEOAE tests were also performed [8,21,26].

When the IA results were analyzed in terms of place of residence, a higher percentage of abnormal results came from rural rather than urban areas. This result is consistent with the literature [23]. The worst IA results in urban children were obtained for those aged 8, whereas in rural areas they were obtained for children aged 9. The otoscopic examination showed abnormalities in the middle ears of all ears that gave an abnormal IA result [8]. Taking both groups together, type B tympanograms were recorded in 24 ears, representing 6.1% of all examined ears. Research in Canada has shown that, in a similar age group, type B tympanograms were seen in 7.8% of tested children [31]. Similarly, in The Netherlands, LeClerque et al. [35] found that 6.3% of children aged 9–11 years had type B or C tympanograms. In our material, type C tympanograms were obtained in 10.5% of all examined ears from both groups. In a study by Czechowicz [8], a problem with highly negative middle ear pressure was found in about 8% of the examined children (aged 6–19 years). Other authors have found much higher values—about 18%—in children aged 6–10 years [24].

Analysis of the PTA results in terms of the place of residence showed that, as was the case with IA, a greater percentage of abnormal results came from rural areas, not urban areas [13,22,36,50]. For urban children, no hearing loss was found in those aged 10–12 years, and, for rural children, in those aged 10. In urban children, the greatest number of hearing impairments was found in 8-year-olds, while in rural areas it was found in 9-year-olds. Taking both groups together, abnormal hearing thresholds were recorded in 25 ears, accounting for 6.4% of all examined ears [13,26,31,34,50]. Some authors have published findings in which this percentage is much higher [3,26,33,36,51,52,53,54,55]. There are a number of possible reasons for the difference, for example, the size of the study group or the season in which the research was conducted. The research here was carried out in the autumn and winter months. A number of the examined children may have had elevated hearing thresholds due to previous infections or taking possibly ototoxic medications (such as antibiotics). Moreover, it has been confirmed in other studies that a greater percentage of abnormal PTA results are found in younger children [21,27,31,55]. This may be because young children are more prone to infections related to obstruction of the Eustachian tube [26,32,53,54,56,57]. Analysis of hearing thresholds in conjunction with IA showed that all cases of hearing loss were related to malfunction of the middle ear. On the other hand, the work of Le Clerq [35] has shown that a type A tympanogram was responsible for 7.8% of the abnormalities, i.e., the pathology was localized to the inner ear.

Analysis of the TEOAE results showed that, as in the two previous tests (PTA and IA), there were significantly more refer results in rural areas than in urban ones. Taking both groups together, the criterion of the presence of a response was not met for 96 ears, which comprised 24.5% of all examined ears, a much higher figure than in other studies [23,31]. This may be related to changes in the middle ear, which were observed in most of these particular children, as we know that even slight middle ear abnormalities have a significant impact on the level of OAE response [58]. Analysis of the TEOAE results in terms of age showed that most “refer” results were found in 12–13-year-old children as well as in 8-year-old children. This finding is not confirmed in the literature. In a study by Feder et al. [31], a higher percentage of “refer” results was found in the younger children (3–5 years old). Moreover, as was shown in the work of Jedrzejczak et al. [59], the SNR in the low and medium frequency range of children aged 6–7 years was 3–5 dB lower than in children aged 11–12 years.

An overall analysis showed that a “refer” result was found in all three tests (AT, AI, TEOAE) for 38 of 197 ears in children from the urban group (Group_u), and in 66 of 195 ears from the rural group (Group_r). Taking both groups together, the greatest number of “refer” results was obtained for TEOAEs, and the lowest for PTA. This may be because school-age children are constantly exposed to noise, which may lower OAE responses before changes are noticeable audiometrically [60,61], especially for very high frequencies (>8 kHz) which are not included in the standard PTA test [62]. Moreover, OAEs are more sensitive to a temporary change in hearing threshold; they also tend to detect more false positives [9,63].

In the present study a screening PTA test was used. Our results show that all cases of childhood hearing loss diagnosed at school age are those with a conductive component. Because the screening audiometer does not allow bone conduction threshold to be evaluated, which allows the type of hearing loss to be determined, the gold standard (next to PTA) should be IA, since it can allow the status of the middle ear to be assessed. That is, an IA test could reduce the time it takes for a child to receive treatment. The IA test can be considered essential, since hearing defects may result in cognitive impairment and learning problems. This is particularly important for rural children, where access to a specialist is more difficult to obtain. Chan et al. [64] point out in their work that people from the country receive hearing aids later than do city residents, explaining it in terms of poorer access to specialists, but also lower socio-economic status. Douthit et al. [10] drew attention to the fact that in rural areas of the USA it is more difficult to keep highly specialized doctors due to lower salaries. According to the literature, the differences between rural and urban areas concern not only hearing test outcomes, but also other health aspects [10,65,66,67,68]. These references are almost unanimous in emphasizing that in rural areas there is poorer access not only to specialists, drugs, and diagnostic equipment, but also to training of medical personnel and lower health awareness.

This study focused on a public health problem that affects a large majority of countries. It is urgent to have a policy to implement screening in different age groups (universal newborn hearing screening, pre-school screening, and school screening) for early detection of hearing loss in children. There are some recommendations, but more action is needed (e.g., [69]). The ideal situation would be for universal hearing screening for children to be performed every few years. It would then reveal not only children who need immediate help but also those with suspicious hearing test results. This might help avoid the uncontrolled development of some conditions, e.g., cholesteatoma [70].

## 5. Conclusions

Acquired hearing impairments in school-age children can result in limited cognitive functions and learning problems. Due to more difficult access to specialists in rural areas, universal hearing screening programs need to detect childhood hearing loss as early as possible and help the child receive prompt treatment. As this research has shown, the best solution would be if the screening tests were based not only on PTA, but also included IA, which allows the condition of the middle ear to be objectively assessed.

Results of the present study confirm prior suggestions that in order to provide equal opportunities for children in rural and city areas, the health system in Poland (and elsewhere around the world) needs to be improved and extended. We suggest that medical personnel in rural areas need better access to training and career development, and that health awareness needs to be increased using appropriate promotional methods. Furthermore, in general, the presence of an audiologist in primary healthcare seems crucial in order to reduce the time taken to diagnose hearing loss in children, and thus promote early intervention.

## Figures and Tables

**Table 1:** Percentage of the type of tympanogram in the examined ears depending on the child’s place of residence. Group_u—urban; Group_r—rural.

	Tympanogram Type (%)		
	A	B	C
Group_u	89.8	4.1	6.1
Group_r	76.9	8.2	14.9

**Table 2:** Percentage of type of tympanogram (A, B, or C) by age and place of residence.

Age (Years)	Group_u Tympanogram Type (%)			Group_r Tympanogram Type (%)		
	A	B	C	A	B	C
8	83	8.5	8.5	94.1	5.9	0
9	86.8	10.5	2.6	54.2	16. 7	29.2
10	90.3	0	9.7	76.7	0	23.3
11	100	0	0	86.2	6.9	6.9
12	100	0	0	79.5	6.8	13.6
13	91.5	0	8.5	92.6	7.4	0

**Table 3:** Percentage of normal hearing threshold (pass) and hearing loss (refer) by age and area of residence.

	Age (Years)					
	8	9	10	11	12	13
Group_u pass	88.9	94.7	100	100	100	95.7
Group_u refer	11.1	5.3	0	0	0	4.3
Group_r pass	94.1	77.1	93.7	100	90.9	96.3
Group_r refer	5.9	22.9	6.3	0	9.1	3.7

**Table 4:** Summary of TEOAE pass and refer percentages according to age.

Age (Years)	TEOAE “Pass” (%)	TEOAE “Refer” (%)
8	78.1	21.9
9	84.3	18.7
10	82.0	18.0
11	81.4	18.6
12	72.9	27.1
13	79.7	20.3

**Table 5:** Summary of the number and percent of ears with “refer” results.

Test	*N* of “Refer” Results in Group_u	*N* of “Refer” Results in Group_r
PTA	6 (3.0%)	19 (9.7%)
IA	20 (10.1%)	45 (23.1%)
TEOAE	34 (17.3%)	62 (31.8%)
PTA or IA	20 (10.1%)	45 (23.1%)
PTA or TEOAE	34 (17.3%)	62 (31.8%)
IA or TEOAE	38 (19.3%)	66 (33.8%)
PTA or IA or TEOAE	38 (19.3%)	66 (33.8%)

## Data Availability

The data that support the findings of this study are available from the first author (E.P.) upon reasonable request.
